# Chenodeoxycholic acid stimulates glucagon‐like peptide‐1 secretion in patients after Roux‐en‐Y gastric bypass

**DOI:** 10.14814/phy2.13140

**Published:** 2017-02-15

**Authors:** Signe Nielsen, Maria S. Svane, Rune E. Kuhre, Trine R. Clausen, Viggo B. Kristiansen, Jens F. Rehfeld, Jens J. Holst, Sten Madsbad, Kirstine N. Bojsen‐Moller

**Affiliations:** ^1^ Department of EndocrinologyCopenhagen University Hospital HvidovreHvidovreDenmark; ^2^ NNF Center for Basic Metabolic ResearchUniversity of CopenhagenCopenhagenDenmark; ^3^ Obesity BiologyNovo Nordisk A/SMaaloevDenmark; ^4^ Department of Surgical GastroenterologyCopenhagen University Hospital HvidovreHvidovreDenmark; ^5^ Department of Clinical BiochemistryCopenhagen University Hospital RigshospitaletCopenhagenDenmark; ^6^ Department of Biomedical SciencesUniversity of CopenhagenCopenhagenDenmark

**Keywords:** Bile acids, GLP‐1, PYY, Roux‐en‐Y gastric bypass

## Abstract

Postprandial secretion of glucagon‐like peptide‐1 (GLP‐1) is enhanced after Roux‐en‐Y gastric bypass (RYGB), but the precise molecular mechanisms explaining this remain poorly understood. Plasma concentrations of bile acids (BAs) increase after RYGB, and BAs may act as molecular enhancers of GLP‐1 secretion through activation of TGR5‐receptors. We aimed to evaluate GLP‐1 secretion after oral administration of the primary bile acid chenodeoxycholic acid (CDCA) and the secondary bile acid ursodeoxycholic acid (UDCA) (which are available for oral use) in RYGB‐operated participants. Eleven participants (BMI 29.1 ± 1.2, age 37.0 ± 3.2 years, time from RYGB 32.3 ± 1.1 months, weight loss after RYGB 37.0 ± 3.1 kg) were studied in a placebo‐controlled, crossover‐study. On three different days, participants ingested (1) placebo (water), (2) UDCA 750 mg, (3) CDCA 1250 mg (highest recommended doses). Oral intake of CDCA increased plasma concentrations of GLP‐1, C‐peptide, glucagon, peptide YY, neurotensin, total bile acids, and fibroblast growth factor 19 significantly compared with placebo (all *P* < 0.05 for peak and positive incremental area‐under‐the‐curve (piAUC)). All plasma hormone concentrations were unaffected by UDCA. Neither UDCA nor CDCA changed glucose, cholecystokinin or glucose‐dependent insulinotropic polypeptide (GIP) concentrations. In conclusion, our findings demonstrate that the primary bile acid chenodeoxycholic acid is able to enhance secretion of gut hormones when administered orally in RYGB‐operated patients—even in the absence of nutrients.

## Introduction

Roux‐en‐Y gastric bypass (RYGB) is an effective treatment of severe obesity (Sjöström 2013; Madsbad et al. 2014) and type 2 diabetes (Madsbad et al. 2014; Schauer et al. 2014). Interestingly, improved glycemia occurs within days after surgery indicating that weight‐loss cannot fully explain improvement in glucose tolerance (Pories et al. 1995). Early improvement in hepatic insulin sensitivity (Bojsen‐Moller et al. 2014), accelerated absorption of nutrients (Dirksen et al. 2013a), altered gut hormone secretion (Jacobsen et al. 2012), and enhanced insulin secretion (Jacobsen et al. 2012) all appear to contribute to the weight loss and improved glucose metabolism seen postoperatively. After RYGB, studies have consistently reported a 7–10 fold enhanced postprandial secretion of glucagon‐like peptide‐1 (GLP‐1), which is of major importance for the improved beta‐cell function in patients with preoperative type 2 diabetes (Jørgensen et al. 2012, 2013) and linked to postoperative weight loss (Dirksen et al. 2012). However, the mechanisms behind the enhanced GLP‐1 secretion after RYGB remain poorly understood.

Several studies have demonstrated increased plasma bile acids (BAs) after RYGB (Nakatani et al. 2009; Jansen et al. 2011; Pournaras et al. 2012; Ahmad et al. 2013; Kohli et al. 2013a; Steinert et al. 2013; Werling et al. 2013; Dutia et al. 2015; Jørgensen et al. 2015), and endogenous BAs have therefore been proposed as key‐mediators of the improvements in glucose metabolism (Pournaras et al. 2012; Kohli and Seeley 2013; Kohli et al. 2013b; Ryan et al. 2014; Baud et al. 2016). Moreover, studies have demonstrated an association between plasma BAs, GLP‐1 and improved glucose tolerance months to years after RYGB (Nakatani et al. 2009; Pournaras et al. 2012; Kohli et al. 2013a; Steinert et al. 2013; Werling et al. 2013; Dutia et al. 2015; Jørgensen et al. 2015). Within the first week after RYGB, GLP‐1 secretion is enhanced and glucose tolerance markedly improved while plasma BAs still seem unchanged (Steinert et al. 2013; Jørgensen et al. 2015), although one study reports increased BAs already 4 days after RYGB (Pournaras et al. 2012). Hence, it remains unclear to what extent endogenous BAs are involved in the exaggerated GLP‐1 and insulin responses after RYGB.

Administration of BAs has been demonstrated to stimulate GLP‐1 secretion in vitro via activation of the TGR5 receptor, which is activated by most BAs (Kawamata et al. 2003; Brighton et al. 2015). A stimulated secretion was also observed in humans after rectal (Adrian et al. 2012; Wu et al. 2013a) and intracolonic infusions (Adrian et al. 1993) in non‐operated healthy volunteers (Adrian et al. 1993; Wu et al. 2013a) and patients with type 2 diabetes (Adrian et al. 2012). In contrast, neither intragastric nor upper intestinal administration of BAs were associated with clinically relevant GLP‐1 responses (Meyer‐Gerspach et al. 2013; Wu et al. 2013b; Hansen et al. 2016), which might suggest that an exposure of the distal small intestine is required for BAs to stimulate robust GLP‐1 secretion. As a consequence of the bypass of stomach and upper small intestine after RYGB, nutrients and other ingested molecules will rapidly reach the distal part of the small intestine where the density of GLP‐1 producing L‐cells is high (Eissele et al. 1992).

The aim of our study was to evaluate whether oral administration of BAs is able to increase GLP‐1 secretion in RYGB‐operated participants. We studied intake of two different BAs that are available as pharmacological agents used for treatment of gallstones: the primary BA chenodeoxycholic acid (CDCA) and the secondary BA ursodeoxycholic acid (UDCA). We chose to use the highest recommended daily dose for each bile acid according to the label, which was 1250 mg for CDCA and 750 mg for UDCA. We hypothesized that oral intake would stimulate GLP‐1 secretion in RYGB‐operated participants.

## Methods

### Participants

Eleven (six males and five females) participants were recruited from Hvidovre Hospital's bariatric surgery outpatient clinic (Table 1). Inclusion criteria were uncomplicated primary RYGB >3 months prior to inclusion and normal glucose tolerance (fasting plasma glucose <7.0 mmol/L and HbA1c <48 mmol/mol), and exclusion criteria were anemia (plasma hemoglobin <6.5 mmol/L), dysregulated hypothyroidism, use of anti‐thyroid medication, renal insufficiency, prior pancreatitis, certain RYGB complications (severe reactive hypoglycemia, dysphagia), pregnancy or lactation. All participants maintained stable weight during the study period. Medications include vitamins (multivitamins, zink, D‐vitamins and B12‐injections), proton‐pump inhibitors and antihypertensive drugs. None of the participants had cholecystectomy prior to inclusion; however, one participant underwent acute cholecystectomy before the last study day due to an acute attack of simple gallstones. One participant only completed the UDCA day and another participant did not complete the CDCA day; moreover, one participant was excluded from data analysis from the CDCA day due to excessively high morning C‐peptide concentrations indicating a non‐fasting state.

**Table 1:** Study participants. Data are presented as mean ± SEM

*n* = 11	
Age (years)	37.0 ± 3.2
Time since RYGB (months)	32.3 ± 1.1
Weight loss (kg)	37.0 ± 3.1
Weight (kg)	90.5 ± 5.5
Body mass index (kg/m^2^)	29.1 ± 1.2
Fasting plasma glucose (mmol/liter)	4.9 ± 0.1
HbA1c (mmol/mol)	33.2 ± 0.9

Written informed consent was obtained from all participants before entering the study. The protocol was approved by the Municipal Ethical Committee of Copenhagen in accordance with the Helsinki II declaration, by the Danish Data Protection Agency and was registered at www.ClinicalTrials.gov (NCT 02340247).

### Study design

Participants were examined on three separate days separated by at least 2 days with the following interventions: **Placebo:** Oral intake of 150 mL water; **UDCA**: Oral intake of UDCA 750 mg (Ursofalk^®^ capsules, Dr. Falk Pharma GmbH, Freiburg, Germany) suspended in 50 mL water followed by oral intake of 100 mL water; **CDCA:** Oral intake of CDCA 1250 mg (Xenbilox^®^ capsules, Juers Pharma ImportExport GmbH, Hamburg, Germany) suspended in 50 mL water followed by oral intake of 100 mL water. Dosages corresponded to the highest recommended daily dose of CDCA and UDCA respectively.

Before the experiments, participants were instructed to fast overnight (10–12 h). On each study day, participants were weighed, a catheter was inserted into a forearm vein and three basal blood samples were drawn (–10, –5 and 0 min). At *t* = 0 placebo, UDCA or CDCA was ingested within 5 min. Blood was sampled frequently for a total of 3 h (at *t* = 5, 10, 15, 20, 25, 30, 45, 60, 90, 120, 180 min). In addition to measurements of blood pressure and heart rate, appetite perception, nausea, and stomach pain were evaluated using visual analogue scale (VAS) scoring at *t* = 0, 30, 60, 120, 180 min.

### Sample collection and laboratory analyses

Blood was collected into clot activator tubes for C‐peptide analysis and into EDTA‐coated tubes for analyses of glucose, GLP‐1, glucagon, PYY, GIP, CCK, neurotensin, total bile acids (TBA) and fibroblast growth factor 19 (FGF19). Clot activator tubes were left at room temperature to coagulate for 30 min, whereas EDTA‐tubes were immediately placed on ice until centrifuged at 4°C (2000*g* for 10 min). Plasma glucose was measured using the glucose oxidase technique (YSI model 2300 STAT Plus; YSI, Yellow Springs, OH). Serum for measurements of C‐peptide was frozen and stored at −80°C and plasma at −20°C until batch analysis.

Serum C‐peptide concentrations were determined using Immulite 2000 analyzer (Siemens Healthcare Diagnostics Inc., Tarrytown, NY, USA). Plasma samples were assayed for total GLP‐1 immunoreactivity using antiserum 89390 as previously described (Ørskov et al. 1994). Glucagon was measured by in‐house radioimmunoassay (RIA) using antibody 4305 (Jørgensen et al. 2012). Total PYY was measured by an in‐house RIA using a monoclonal antibody MAB8500 as described elsewhere (Toräng et al. 2016).

Total GIP was measured by in‐house RIA using antibody 80867 directed against the C‐terminal region (Jørgensen et al. 2012). CCK was measured by RIA using antiserum no. 92128, which binds all the bioactive forms of CCK in circulation (CCK‐58, ‐33, ‐22 and ‐8) with equal potency, and without cross‐reactivity with homologous gastrin peptides (Rehfeld 1998). Neurotensin was measured by in‐house RIA employing N‐terminal directed antiserum (no. 3D97), thus measuring total neurotensin (Kuhre et al. 2015). TBA concentrations were determined using an enzyme cycling based TBA Assay Kit from Diazyme (Cat No DZ042A‐K, San Diego, CA, USA; intra‐assay coefficient of variation (CV): 3.9%, inter‐assay CV: 2.9%). FGF19 concentrations were determined using a commercial human FGF19 sandwich ELISA kit from BioVendor (Cat No RD191107200R, BioVendor, Brno, Czech Republic; intra‐assay CV: 6.0%; inter‐assay CV: 7.5%).

### Statistical analyses and calculations

Basal concentrations were calculated as the mean of the three time points (*t* = −10, −5 and 0 min). Positive incremental area‐under‐the‐curve (piAUC) was calculated using the trapezoid rule as the AUC above basal concentrations to assess postprandial secretions. Data are expressed as mean ± SEM.

Differences between the three study days were analyzed by ANOVA in a linear mixed effects model with study day (placebo, UDCA, CDCA) as fixed effect and individual participants as random effect. Post hoc testing was performed comparing CDCA and UDCA with placebo if ANOVA revealed significant differences between study days. Statistical analysis was performed in R version 3.1.2 (www.R-projec.org) with the package “nlme” for the linear mixed effects models. *P* < 0.05 were considered statistically significant.

## Results

### Basal hormone concentrations

Basal hormone concentrations did not differ between study days (Table 2).

**Table 2:** GLP‐1, PYY, neurotensin, glucose, C‐peptide, CCK, and GIP concentrations in response to bile acid administration

	Placebo *n* = 10	CDCA *n* = 8	UDCA *n* = 11	ANOVA	Placebo versus CDCA	Placebo versus UDCA
Basal GLP‐1 (pmol/L)	12.7 ± 0.9	14.3 ± 1.5	12.2 ± 1.3	*P = *0.25		
Peak GLP‐1 (pmol/L)	18.0 ± 2.2	29.1 ± 2.2	21.8 ± 3.2	*P = *0.01	*P < *0.01	*P = *0.18
piAUC GLP‐1 (pmol/L × min)	226 ± 105	815 ± 104	444 ± 102	*P < *0.01	*P < *0.01	*P = *0.14
Basal PYY (pmol/L)	11.4 ± 0.8	8.9 ± 2.2	10.2 ± 1.0	*P = *0.44		
Peak PYY (pmol/L)	12.9 ± 0.8	18.5 ± 1.6	14.0 ± 1.0	*P = *0.01	*P < *0.01	*P = *0.48
piAUC PYY (pmol/L × min)	28.8 ± 12.0	937 ± 224	263 ± 79.9	*P < *0.01	*P < *0.01	*P = *0.17
Basal neurotensin (pmol/L)	16.2 ± 3.1	15.7 ± 3.6	15.5 ± 1.9	*P = *0.77		
Peak neurotensin (pmol/L)	21.9 ± 4.7	66.9 ± 10.0	30.0 ± 4.6	*P < *0.01	*P < *0.01	*P = *0.16
piAUC neurotensin (pmol/L × min)	123 ± 45.2	2399 ± 418	683 ± 371	*P < *0.01	*P < *0.01	*P = *0.16
Basal glucose (mmol/L)	5.0 ± 0.1	4.9 ± 0.1	5.0 ± 0.13	*P = *0.58		
Peak glucose (mmol/L)	5.1 ± 0.1	5.2 ± 0.1	5.1 ± 0.15	*P = *0.98		
piAUC glucose (mmol/L × min)	9.7 ± 3.6	14.6 ± 4.6	11.5 ± 7.9	*P = *0.87		
Basal C‐peptide (pmol/L)	548 ± 52.7	483 ± 43.1	485 ± 41.1	*P = *0.33		
Peak C‐peptide (pmol/L)	592 ± 54.2	624 ± 59.2	557 ± 44.9	*P = *0.54		
piAUC C‐peptide (pmol/L × min)	1716 ± 604	7094 ± 2506	3119 ± 1234	*P = *0.03	*P = *0.01	*P = *0.41
Basal glucagon (pmol/L)	7.4 ± 0.8	8.0 ± 1.4	7.5 ± 1.1	*P = *0.93		
Peak glucagon (pmol/L)	9.1 ± 1.2	13.8 ± 2.4	9.9 ± 1.6	*P < *0.01	*P < *0.01	*P = *0.41
piAUC glucagon (pmol/L × min)	32.8 ± 11.6	283 ± 58.3	60.3 ± 23.1	*P < *0.01	*P < *0.01	*P = *0.52
Basal CCK (pmol/L)	0.5 ± 0.1	0.5 ± 0.1	0.5 ± 0.1	*P = *0.30		
Peak CCK (pmol/L)	1.3 ± 0.2	1.8 ± 0.4	1.1 ± 0.2	*P = *0.05	*P = *0.06	*P = *0.49
piAUC CCK (pmol/L × min)	16.5 ± 4.9	24.1 ± 6.3	21.8 ± 6.1	*P = *0.57		
Basal GIP (pmol/L)	10.8 ± 0.7	10.8 ± 0.8	9.9 ± 1.1	*P = *0.61		
Peak GIP (pmol/L)	13.6 ± 1.0	14.4 ± 0.5	12.8 ± 0.6	*P = *0.37		
piAUC GIP (pmol/L × min)	113 ± 39.3	162 ± 82.4	169 ± 80.9	*P = *0.74		

CDCA, chenodeoxycholic acid; UDCA, ursodeoxycholic acid. Data are presented as mean ± SEM.

### Primary outcome: GLP‐1 secretion

**Figure 1:**
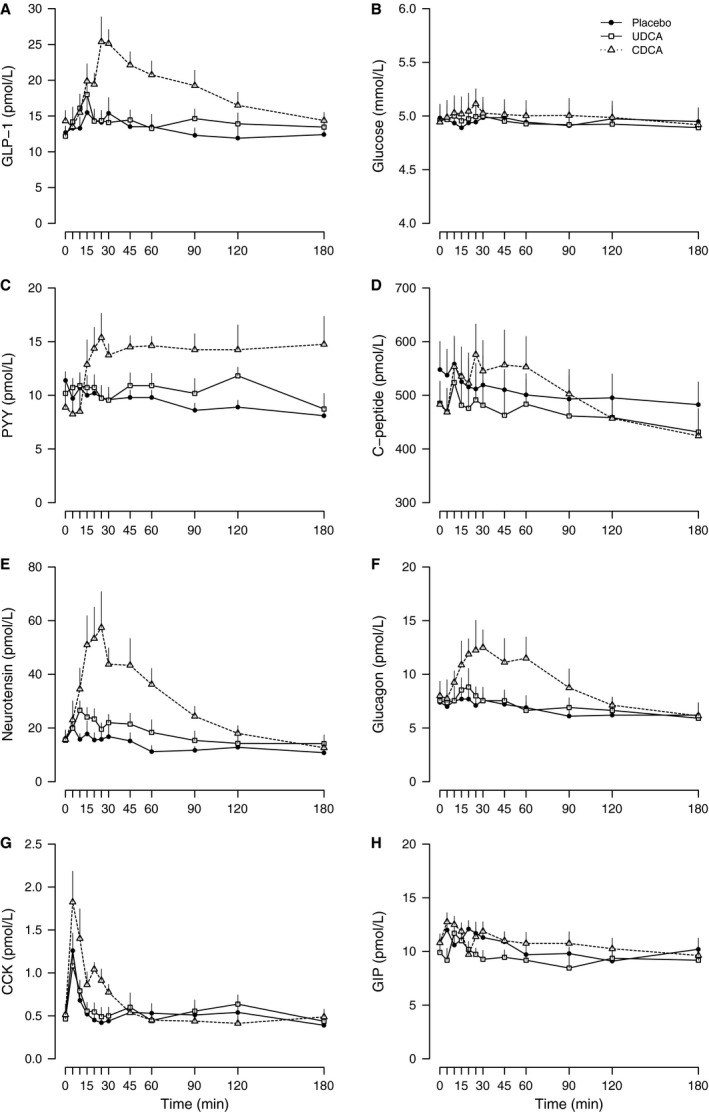
GLP‐1 (A), glucose (B), PYY (C), C‐peptide (D), neurotensin (E), glucagon (F), CCK (G), and GIP (H) concentrations in response to oral intake of placebo (solid line, black circles), ursodeoxycholic acid (UDCA; solid line, white squares) and chenodeoxycholic acid (CDCA; dotted line, white triangles) in RYGB‐operated participants. Data are presented as mean + SEM.

GLP‐1 secretion differed significantly between study days (ANOVA: piAUC *P* < 0.01 and peak *P* = 0.01). Oral intake of CDCA increased GLP‐1 secretion from a basal of 14.3 ± 1.5 pmol/L to a mean peak concentration of 29.1 ± 2.2 pmol/L (*P* < 0.01 vs. placebo), whereas GLP‐1 concentrations were unaffected by UDCA (Table 2, Fig. 1A). One participant underwent cholecystectomy before the CDCA day, but GLP‐1 responses (piAUC: 1032 pmol/L × min, peak: 38 pmol/L) on this day did not differ markedly from the overall mean response to CDCA.

### Plasma glucose, serum C‐peptide, and plasma glucagon

Plasma glucose concentrations did not differ between study days (Table 2, Fig. 1B). C‐peptide secretion differed between study days (ANOVA: piAUC *P* = 0.03) and was slightly increased after oral intake of CDCA (*P* = 0.01) in contrast to an absent response after UDCA compared with placebo. However, peak C‐peptide concentrations were not significantly affected (ANOVA: peak *P* = 0.54; Table 2, Fig. 1D). Glucagon secretion also differed between study days (ANOVA: *P* < 0.01 for both piAUC and peak) with increased secretion following CDCA but no effect of UDCA (Table 2, Fig. 1F).

### Other gut hormones

PYY secretion differed between study days (ANOVA: *P* ≤ 0.01 for both piAUC and peak) with increased secretion after CDCA compared with placebo, whereas UDCA did not stimulate PYY secretion (Table 2, Fig. 1C). Intake of CDCA resulted in a prolonged PYY secretion, i.e. concentrations did not return to basal level within 180 minutes after intake (*P* = 0.02). Secretion of neurotensin also differed between study days (ANOVA: *P* < 0.01 for both piAUC and peak) with clearly enhanced secretion after CDCA in contrast with no stimulation during UDCA compared with placebo (Table 2, Fig. 1E). GIP was not affected by any of the two BAs (Table 2, Fig. 1H). Peak of CCK concentrations (ANOVA: *P* = 0.05) tended to increase after CDCA (*P* = 0.06) but not after UDCA (*P* = 0.49) compared with placebo, while piAUC was unaffected by both BAs (ANOVA: *P* = 0.57; Table 2, Fig. 1G).

### Plasma TBA and FGF19

**Figure 2:**
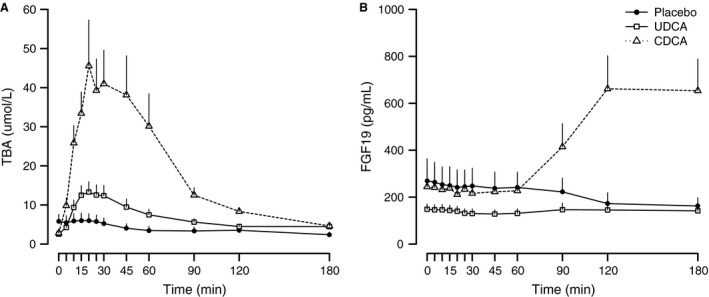
Total bile acids (TBA) (A) and fibroblast growth factor 19 (FGF19) (B) concentrations in response to oral intake of placebo (solid line, black circles), ursodeoxycholic acid (UDCA; solid line, white squares) and chenodeoxycholic acid (CDCA; dotted line, white triangles) in RYGB‐operated participants. Data are presented as mean + SEM.

Both plasma TBA and FGF19 differed between study days (ANOVA: *P* < 0.01 for both piAUC and peak). CDCA increased plasma TBA significantly, while only a slight numerical increase in piAUC (*P* = 0.07) and no change in peak concentrations (*P* = 0.41) were observed following UDCA (Table 3, Fig. 2A) compared with placebo. FGF19 concentrations increased after CDCA compared with placebo (*P* ≤ 0.01 for both piAUC and peak) and were unchanged following UDCA (*P* = 0.91 for piAUC, *P* = 0.16 for peak; Table 3, Fig. 2B).

**Table 3:** Total bile acid (TBA) and fibroblast growth factor 19 (FGF19) concentrations in response to bile acid administration

	Placebo *n* = 10	CDCA *n* = 8	UDCA *n* = 11	ANOVA	Placebo vs. CDCA	Placebo vs. UDCA
Basal TBA (*μ*mol/L)	5.8 ± 1.8	2.8 ± 0.6	2.6 ± 0.8	*P = 0.08*		
Peak TBA (*μ*mol/L)	7.1 ± 1.8	54.0 ± 12.5	13.9 ± 2.6	*P < 0.01*	*P < 0.01*	*P = 0.41*
piAUC TBA (*μ*mol/L × min)	32.1 ± 14.8	2844 ± 567	767 ± 139	*P < 0.01*	*P < 0.01*	*P = 0.07*
Basal FGF19 (pg/mL)	270 ± 93.9	244 ± 91.3	149 ± 22.1	*P = 0.26*		
Peak FGF19 (pg/mL)	304 ± 90.2	776 ± 153	177 ± 31.8	*P < 0.01*	*P = 0.01*	*P = 0.16*
piAUC FGF19 (pg/mL × min)	2104 ± 910	37174 ± 9049	1477 ± 926	*P < 0.01*	*P < 0.01*	*P = 0.91*

CDCA, chenodeoxycholic acid; UDCA, ursodeoxycholic acid. Data are presented as mean ± SEM.

### VAS ratings, blood pressure, and heart rate

Oral intake of BAs neither changed VAS ratings for appetite perception (hunger or satiety), nausea, or stomach pain nor affected blood pressure or heart rate (data not shown). However, one patient did report increased VAS (64 mm from baseline) in stomach pain 30 min after intake of CDCA. This was followed by a single defecation after which VAS returned to baseline.

## Discussion

We investigated the effects of oral administration of two different bile acids (BAs), ursodeoxycholic acid (UDCA) and chenodeoxycholic acid (CDCA) in RYGB‐operated participants.

We found that administration of the primary BA, CDCA, stimulated secretion of GLP‐1, PYY, neurotensin, C‐peptide and glucagon, whereas plasma glucose, CCK and GIP concentrations were unaffected. In contrast, ingestion of the secondary BA, UDCA, did not affect any hormone concentrations significantly. Interestingly, CDCA induced peak GLP‐1 concentrations of 29 pmol/L and notably, this was obtained without concomitant intake of nutrients or calories. A GLP‐1 response of this size corresponds to the normal GLP‐1 secretion during a liquid mixed‐meal in non‐operated obese subjects (Jørgensen et al. 2012) and is much higher than previously reported after upper intestinal infusions of BAs (1250 mg CDCA (Hansen et al. 2016), 2100 mg CDCA (Meyer‐Gerspach et al. 2013), or 2000 mg taurocholic acid (TCA) (Wu et al. 2013b)), where maximal mean peak GLP‐1 concentrations reached 17 pmol/L (Hansen et al. 2016). Studies with rectal administrations of BAs (TCA) have shown a more pronounced GLP‐1 secretory response with peak concentrations up to ~35 pmol/L (Adrian et al. 2012; Wu et al. 2013a). After RYGB, passage of orally ingested substances is accelerated through the gastric pouch that drains directly into the lower part of the jejunum (Dirksen et al. 2013a); accordingly, oral intake of BAs corresponds to administration directly into distal parts of the small intestine. Thus, our findings add to the existing literature and support that GLP‐1 secretion after BA administration depends on the site of delivery. This may be particularly relevant for unconjugated BAs, which normally will be passively absorbed in the upper intestine before reaching the distal small intestine after oral administration in non‐RYGB individuals (Ma and Patti 2014).

In RYGB‐operated patients, however, GLP‐1 secretion may reach peak concentrations up to ~150 pmol/L after a liquid mixed meal (Jørgensen et al. 2012), hence CDCA does not seem to activate the secretory machinery of the L‐cell to its full capacity—at least not when BAs are ingested alone without nutrients. Nevertheless, the GLP‐1 secretion after CDCA was associated with a small increase in C‐peptide concentrations even without co‐ingestion of nutrients and without changes in glucose. Whether this insulin secretion was actually mediated by GLP‐1 cannot be determined from this study, but if so, we would not expect a major response given that the insulinotropic effect of GLP‐1 is glucose‐dependent (Hvidberg et al. 1994).

Administration of BAs has been demonstrated to stimulate PYY in vitro via activation of TGR5 (Bala et al. 2014). Stimulation also appears to occur in vivo (Adrian et al. 1993, 2012; Meyer‐Gerspach et al. 2013; Wu et al. 2013a), as demonstrated after intraduodenal (Meyer‐Gerspach et al. 2013), intracolonic (Adrian et al. 1993), and rectal (Adrian et al. 2012) infusions of BAs (CDCA, deoxycholate (DCA) and TCA, respectively), and further supported by our findings of an increase in PYY concentrations after oral intake of CDCA. Notably, markedly higher peak concentrations of PYY have been reported after ingestion of mixed meals in both non‐operated subjects and RYGB‐operated patients (Jacobsen et al. 2012) as well as after rectal administration of BAs (Adrian et al. 2012; Wu et al. 2013a). Neurotensin appears to be a co‐player with GLP‐1 and PYY in regulating metabolic functions, including appetite, and glucose homeostasis regulation (Svendsen et al. 2015; Grunddal et al. 2016) and as for GLP‐1 and PYY, RYGB is associated with greatly exaggerated postprandial secretion of this peptide (Dirksen et al. 2013b). We found that administration of CDCA stimulated secretion of neurotensin and the secretory profile was rather similar to that of GLP‐1. Interestingly, the secretory profiles of GLP‐1 and neurotensin appeared to differ from the profile of PYY on the CDCA day, with GLP‐1 and neurotensin returning to baseline level after 120–150 min, whereas PYY levels remained high throughout the 180 min follow‐up. The present observation is in contrast to the parallel dynamic changes of the hormones often observed during a mixed meal test (Jacobsen et al. 2012). A similar prolonged secretion of PYY has not been reported previously and caused us to speculate that CDCA may, in succession, first stimulate the L‐cells in the proximal ileum followed by stimulation of the L‐cells in the distal part of the intestine including the colon. Indeed, there is data to suggest that the L‐cells in the distal part of the gut primarily secrete PYY, as demonstrated in several animal models (Svendsen et al. 2015; Grunddal et al. 2016; Wewer Albrechtsen et al. 2016). Our results suggest that CDCA is able to simultaneously enhance the secretion of several enteroendocrine cell products (GLP‐1, neurotensin, PYY), which could have synergistic beneficial actions with respect to appetite inhibition (Schmidt et al. 2016).

We observed increased concentrations of glucagon only after CDCA intake, which is in line with previous observations (Meyer‐Gerspach et al. 2013; Hansen et al. 2016). It is well established that RYGB‐operated patients have increased postprandial glucagon concentrations concomitantly with high glucose, GLP‐1 and insulin concentrations (Jacobsen et al. 2012; Jørgensen et al. 2013; Svane et al. 2016). The cause of this paradoxical hyperglucagonemia is still unknown, but extrapancreatic secretion may occur, as observed after pancreatectomy using Whipple procedure (Holst et al. 1983; Lund et al. 2016), which implies similar gastrointestinal reconstructions as after RYGB. Another explanation of our findings could be a direct stimulatory effect of CDCA on the alpha‐cells in pancreas. However, in contrast with the case for CDCA‐stimulated GLP‐1 secretion (Brighton et al. 2015), activation of TGR5 is unlikely be involved as the receptor has been found not to be expressed in primary murine alpha‐cells (Kuhre et al. 2016). BA administration did not change GIP or CCK concentrations in our study, although there was a tendency for slightly increased peak concentrations of CCK after CDCA. In line with these results, other studies have demonstrated increased CCK after intraduodenal (Meyer‐Gerspach et al. 2013) and intragastric (Hansen et al. 2016) CDCA infusions and no effect on GIP concentrations (Hansen et al. 2016).

In this study, we found a marked difference in gut hormone responses between administration of CDCA (1250 mg) and UDCA (750 mg). As this study was an exploratory study of CDCA and UDCA in RYGB‐operated participants, we chose to use the highest recommended daily dose for each bile acid according to the label, which was 1250 mg for CDCA and 750 mg for UDCA. The study was therefore not designed to compare the relative effects of CDCA and UDCA on gut hormone secretion, but rather to investigate whether these BAs stimulated gut hormone secretions at all. Dose‐response studies of different BAs with respect to GLP‐1 secretion have not been performed in humans, but an in vitro study has demonstrated that CDCA activates TGR5 while UDCA was without effect (at equal concentrations) (Kawamata et al. 2003). Differences in the absorption of the two BAs could also be of importance for the ability to stimulate release of gut hormones. In a recent study, BAs activated basolateral but not apically located TGR5 receptors, indicating that BAs must be absorbed from the intestinal lumen before they can stimulate GLP‐1 secretion (Brighton et al. 2015). In addition to the acute TGR5 mediated effects, BAs may have additional and potentially longer lasting effects via activation of the nuclear receptor FXR (Ma and Patti 2014). Activation of FXR regulates insulin secretion (Renga et al. 2010) and induces expression and secretion of FGF19, which stimulates glycogen synthesis and reduce hepatic gluconeogenesis (Ma and Patti 2014). Knockout of FXR in mice eliminated the beneficial effects of sleeve gastrectomy on glucose metabolism (Ryan et al. 2014). Activation of FXR does not seem to have stimulatory effects on GLP‐1 secretion (Brighton et al. 2015) and may even decrease secretion (Trabelsi et al. 2015). CDCA is the most potent ligand for stimulating and activating FXR, while UDCA has negligible FXR‐activities (Ma and Patti 2014). Whether a single administration of BAs can activate the FXR‐pathway is unknown, but in this study we demonstrated that CDCA increased FGF19 secretion after 90 min, although we do not know whether this was indeed a result of FXR pathway activation.

After RYGB, plasma BA concentrations increase both in the fasting state and postprandially (Pournaras et al. 2012; Kohli et al. 2013a; Steinert et al. 2013; Werling et al. 2013; Jørgensen et al. 2015). Endogenously secreted BAs in humans consist mostly of the primary BAs, cholic acid (CA) and CDCA and the secondary BA, DCA; most BAs are conjugated with glycine and the remaining with taurine (Werling et al. 2013; Ma and Patti 2014; Jørgensen et al. 2015). Studies in rodent models strongly support BAs as key mediators of the improvements in glucose metabolism after RYGB and sleeve gastrectomy (Pournaras et al. 2012; Kohli and Seeley 2013; Kohli et al. 2013b; Ryan et al. 2014; Baud et al. 2016), but inconsistencies with respect to timing of the postoperative changes in BAs and the effects of RYGB on GLP‐1 secretion and glucose metabolism are observed in human studies (Ahmad et al. 2013; Steinert et al. 2013; Dutia et al. 2015; Jørgensen et al. 2015). Notably, administration of exogenous BAs causes much higher plasma TBA concentrations (obviously depending of the BA dosage) than seen in RYGB‐operated patients. In our study, CDCA administration increased TBA concentrations up to 5–fold with peak concentrations of 55 *μ*mol/L. In comparison, postprandial TBA concentrations after RYGB reach peak concentrations of only 8–9 *μ*mol/L 12–15 months postoperatively (Werling et al. 2013; Jørgensen et al. 2015). Thus, despite our findings of increased GLP‐1 secretion in RYGB‐operated participants in response to pharmacological doses of CDCA, we cannot make any conclusions regarding the possible physiological effects of endogenous BAs for improved glucose metabolism after RYGB, which obviously is of great interest. The use of only one dose of each BA, and two different doses of BAs, further limit the conclusions with respect to relative effects of CDCA versus UDCA on gut hormone secretion. Another interesting research question is whether exogenous administered BAs can be used to enhance endogenous gut hormone secretion in combination with meal intake in obese and RYGB‐operated patients. If so, BA administration could be a potential treatment for patients with obesity in general, and in particular RYGB‐operated patients with inadequate glycemic control or poor weight loss responses after surgery.

In conclusion, oral administration of the bile acid, chenodeoxycholic acid, to RYGB‐operated participants stimulated GLP‐1, PYY, neurotensin, C‐peptide, and glucagon secretion in the absence of nutrients and without changed glucose concentration. No effect was seen following ursodeoxycholic acid. Chenodeoxycholic acid may therefore be viewed as a molecular enhancer of several gut hormones of potential importance for the future treatment of diabetes and obesity.

## Conflict of Interest

TRC works for Novo Nordisk Denmark and is a minor stockholder in Novo Nordisk A/S and Zealand Pharma A/S. All other authors have reported no relevant conflicts of interest.
